# Effect of Fragrant Primula Flowers on Physiology and Psychology in Female College Students: An Empirical Study

**DOI:** 10.3389/fpsyg.2021.607876

**Published:** 2021-02-23

**Authors:** Songlin Jiang, Li Deng, Hao Luo, Xi Li, Baimeng Guo, Mingyan Jiang, Yin Jia, Jun Ma, Lingxia Sun, Zhuo Huang

**Affiliations:** College of Landscape Architecture, Sichuan Agricultural University, Chengdu, China

**Keywords:** fragrance stimulation, Primula, electroencephalogram, blood pressure, pulse rate, psychological questionnaire

## Abstract

Indoor plants can positively impact physical and mental health in daily life. However, the benefits of viewing indoor plants may be enhanced if the plants emit a fragrant aroma. In this crossover-design study, we measured the physiological and psychological effects of fragrant and non-fragrant Primula plants on 50 female college students, and explored whether aroma stimulation had additive benefits for this group. Non-fragrant *Primula malacoides Franch* was used as a control stimulus, and *Primula forbesii Franch*, which has a floral fragrance, was used as an experimental stimulus. We measured blood pressure, pulse rate, and electroencephalogram (EEG) to evaluate physiological responses, and used a mood state profile and the semantic differential (SD) method to evaluate psychological responses. We found that mean blood pressure and pulse rate decreased significantly after the experiment in both conditions. EEGs showed that the mean values of high alpha waves, high beta waves, and relaxation scores were significantly higher in the experimental vs. control condition. The average scores on each subscale of the psychological questionnaire improved after the experiment in both conditions, and the vitality (V) subscale and total emotional state scores were significantly better in the experimental vs. control condition. The results of the SD method showed that the sense of relaxation and comfort were significantly higher in the experimental vs. control condition. Compared with the non-fragrant Primula, the fragrant Primula induced relatively better physiological and psychological effects.

## Introduction

Since the turn of the 20th century, as electronic information technology has been leaping forward, people are enabled to work and get entertained by more significantly relying on the machine environment (Reyns et al., 2011). The main living environment of modern society has transformed from outdoors to indoors, so individual daily activities are primarily generated indoors (Chang and Chen, 2005; Gong et al., 2012; Linjing and Qihong, 2018). Poor air quality and limited opportunities for physical activity in indoor environments have been associated with a decrease in work efficiency as well as various physiological and psychological conditions (Gullone, 2000; Watts et al., 2015; Reichert et al., 2019). Therefore, the deliberate construction of improved indoor environments has been a subject of increasing focus by researchers (Flies et al., 2019).

Previous theories such as the Pro Nature Theory (Kellert, 1995), Attention Recovery Theory (Kaplan, 1995), and Stress Relief Theory (Ulrich et al., 2008) all state that the natural environment plays an important role in promoting human physical and psychological health. Although plants are generally found in parks and other public green spaces, indoor plants represent a natural resource that can be low-cost and more convenient to access for urban residents. Indoor plants can effectively increase contact time and intimacy with nature, and thereby promote physical and psychological health (Bringslimark et al., 2009; Deng and Deng, 2018).

Most existing research on the impact of indoor plants on human beings has focused on the visual perception of plants. Previous studies have confirmed that visual contact with indoor plants can increase relaxation, relieve stress (Ulrich, 1986; Bringslimark et al., 2009), improve work efficiency (Evensen et al., 2015), enhance work and life satisfaction (Dravigne et al., 2008; Qin et al., 2014), and even relieve pain (Park et al., 2004).

However, many plants, especially ornamental plants, have aromatic characteristics (Haviland-Jones et al., 2005; Bushdid et al., 2014). Fragrant plants can represent a vital natural resource and impact physical and psychological health *via* olfactory channels (Lorig et al., 1990; Jo et al., 2013; Glass et al., 2014; Swamy and Sinniah, 2015). For example, the aroma of rose essential oil has been found to induce physiological and psychological relaxation (Kim et al., 2016), and the aromas of flowers and plants have been found to positively influence patients who have undergone surgery, even reducing the intake of postoperative analgesics (Park and Mattson, 2009).

As a research method for examining the effect of indoor plant aromas on human recovery, psychological questionnaires have been widely used (Ulrich et al., 2008). Since the physiological responses of humans to environmental stimuli may exceed their subjective self-cognition, it is difficult to objectively measure these physiological and emotional responses using psychological questionnaires. Therefore, some scholars have proposed a new research method in which brain waves are used to represent emotional state (Niedermeyer and Silva, 2004). Electroencephalogram (EEG) refers to the recording of electrical signals of the human brain at a scalp level (Lina and Karwowski, 2020). Under the active brain, the postsynaptic potentials of pyramidal neurons will be hyperpolarized and depolarized, and EEG signals are generated. Specific to this method, sensors are placed on the scalp surface to record electrophysiological signals generated by brain activities, which are considered to objectively indicate human emotion changes at a physiological level. The mentioned electrical signals fall to the frequency bands, i.e., delta (0–4 Hz), theta (4–8 Hz), alpha (8–12 Hz), and beta (12–30 Hz). Human behavior, thoughts, and emotions can change the activity of different frequencies of brain waves. Alpha and beta waves are considered to be most closely related to human emotions. Alpha waves reflect a relatively calm and relaxed state, while beta waves correspond to lucidity and quick thinking (Lagopoulos et al., 2009; Alarcao and Fonseca, 2017). An EEG study revealed that the aroma of Japanese plum blossoms reduced brain waves associated with negative emotions and memory impairment (Jo et al., 2013). Further, contact with natural plants was found to enhance alpha and beta waves, reflecting a reduction in mental stress (Hassan et al., 2018a). Experiments by Kim et al. (2016) revealed that an orchid scent produced stronger alpha EEG waves than did a rose aroma, indicating that the orchid scent induced greater feelings of tranquility and relaxation. However, relatively few studies have investigated the physiological effects of aromas using EEG.

At present, most studies examining the effect of plant aromas on humans have used essential oils or perfumes instead of actual plant materials (Jo et al., 2013; Ali et al., 2015; Kim et al., 2016). However, we mainly experience plant odor through exposure to live plants in daily life. To address this in the present study, we exposed participants to live plants, and carried out comparative experiments regarding the most common forms of human-plant contact. As opposed to the mentioned, existing studies using live plants tended to primarily compare the effects of different experimental stimuli, or draw a comparison of the effect of an experimental stimulus vs. a control. Commonly, these studies inadvertently determined the joint effect of vision and smell, in which the effect of olfaction was not distinguished (Adachi et al., 2000; Park and Mattson, 2009; Qin et al., 2014). When participants are subjected to visual stimulation with live plants, the additional effect of aroma is not generally measured. However, this is an important consideration in terms of the role of aromatic stimulation in plant-induced health benefits. Therefore, in the present study, the additional effects exerted by aromatic and non-aromatic plants were compared to determine the effects of fragrant Primula flowers on physiology and psychology in female college students. We measured physiological data including pulse rate, blood pressure, and brain waves, as well as psychological data collected *via* questionnaire.

We had two hypotheses: (1) The exposure to fragrant Primula and non-fragrant Primula will help female college students gain better physiological and psychological states; (2) Fragrant Primula, as compared with non-fragrant Primula, will more significantly enhance physiological and psychological states of female college students.

The present study aims to explore effects of fragrant and non-fragrant Primula flowers on physiological and psychological states of female college students, as well as the additional effect exerted by the fragrance of Primula.

## Materials and Methods

### Participants

In Chinese college dormitory, considerable female students like to cultivate small indoor plants for leisure and decompression. As indicated from existing studies, females are usually more sensitive to odors than males in their daily lives (Camille et al., 2013). To determine the physiological and psychological effects of plant aroma in depth, this study recruited 50 female college students with an average age of 22.32 ± 2.56 years, an average height of 161.58 ± 5.56 cm, and an average weight of 54.05 ± 7.64 kg. Although our sample was relatively homogenous, which could limit the generalizability of our results, this approach was advantageous in that we did not expect to observe differences in olfactory responses resulting from gender, age, or culture. The selection criteria for the participants included: (1) normal olfactory function, no recent history of a cold, sinusitis, or other similar conditions; (2) good physical and psychological health; and (3) no recent use of recreational or pharmaceutical drugs. In addition, within the 48 h before the experiment, participants were asked to abstain from several odorous stimulus (e.g., alcohol, tobacco, caffeine, and perfume).

All participants were informed regarding the content of the experiment, and were told that they had the right to withdraw from the experiment at any time. All participants completed an informed consent form. All study procedures were carried out in accordance with the ethical standards of the National Research Council, and were in accordance with the declaration of Helsinki.

### Materials

Primula is very popular among indoor ornamental plants. It is one of the top-selling indoor potted flowering plants in Europe, America, and East Asia because of it produces colorful flowers early in the season. We selected two kinds of Primula in this experiment. The first was *Primula malacoides Franch*, which is a native Chinese variety with a long history of origin. It was introduced into Europe at the end of the 19th century and has been widely cultivated all over the world, with many horticultural varieties. This kind of Primula has no fragrance (Karlsson, 2001). The second was *Primula forbesii Franch*, which is a wild flower species widely distributed in Southwest China. Primula flowers exhibit a bright color and high ornamental value. Moreover, it can be easily cultivated in a large scale and maintained. It produces a moderate amount of flowers, which have a fresh and elegant fragrance (Hayta et al., 2016). We determined these two kinds of Primula to be suitable materials for testing the effects of aromatic plant stimulation.

### Laboratory Environment

**Figure 1:**
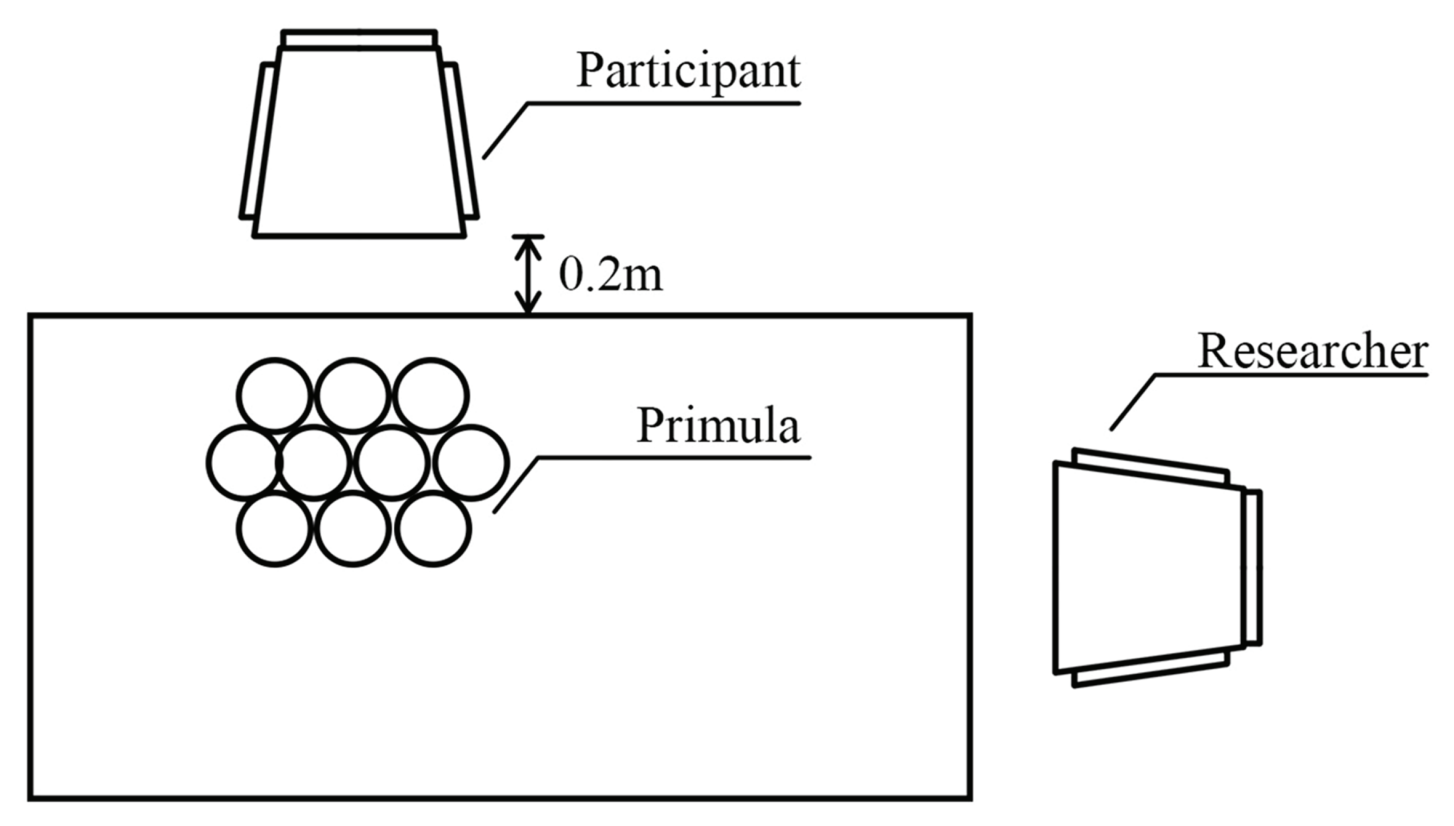
Diagram of the physical set up in relation to the participant.

To ensure strict control the experimental variables, the environmental settings of Ikei et al. (2014) and Hassan et al. (2018a) were referenced and two laboratories with the same indoor layout were selected as the experimental sites for the control condition and the experimental condition. Next to the two laboratories, preparation rooms were arranged in which to measure participant physiological and psychological data before the experiment began. The ceiling and walls of the two laboratories were white, with no decoration. During the experiment, the windows and curtains in each laboratory were kept closed to reduce external sound and light interference. Ten flowerpots with Primula were arranged on the table to ensure that they took up the major field of vision of the participants. Participants were seated in a chair 0.2 m away from the table to closely view and smell the plants, and the distance could be regulated by complying with the participants’ height (Figure 1). During the experiment, the participants were asked to maintain a sitting posture, to relax, and to breathe evenly. The participants in the control condition were exposed to *Primula malacoides Franch*. The participants viewed the plants and did not receive any olfactory stimulation. Those in the experimental condition were exposed to *Primula forbesii Franch*, which has a natural fragrance. The participants viewed the plants, while calmly inhaling the flower fragrance. During the experiment, the room temperature was kept at 25°C and the relative humidity was kept at 60%. Thus, the concentration and transmission of the flower fragrance did not change significantly with time.

### Measurement Items

The experimental data were divided into physiological data and psychological data. Physiological data included pulse rate (BPM), blood pressure [systolic (mmHg), diastolic (mmHg)], and brain waves. It is generally considered that alpha wave and beta wave display the closest relationship to human emotions, in which high alpha wave (about 10–12 Hz) is closely correlated with relaxation, and high beta wave (about 21–30 Hz) corresponds to more attention and alertness (Coben et al., 2010; Bălan et al., 2019). By employing the methods of relevant literature (Hassan et al., 2018a,b), high alpha and high beta waves were analyzed here. Blood pressure and pulse rate were measured two times using a sphygmomanometer on the left arm (Omron, hem-7011, China). We used a Neurosky mindwave EEG headset (Beijing Oriental Creation Technology Co., Ltd., China) to measure EEG signals transmitted from the forehead. The headset had four essential components: (1) a headband, (2) an ear clip, (3) a sensor arm containing the EEG electrode, and (4) a Bluetooth device. The device was light and compact, and did not cause obvious discomfort to users. The device continuously recorded brain wave data and mean value per minute was reported. We used e-Sense™ software for EEG data processing. Specifically, brain wave signals during states of relaxation and attention can be sorted from weak to strong on a scale from 1 to 100 by this software (Sezer et al., 2017). Psychological data included the profile of mood states (POMS) and the semantic differential (SD) method. The POMS includes 40 items, with seven subscales: tension (T), anger (A), fatigue (F), depression (D), vitality (V), confusion (C), and self-esteem (E). Total Mood Disturbance (TMD) was calculated according to the scores from each subscale, with lower TMD scores reflecting a better overall emotional state. The SD method can be used directly quantify the subjective perception of external stimuli. In this study, we selected three adjective items, i.e., comfortable, fascinated, and relaxed, to examine participant psychological responses. Participant scores were calculated according to the content of verbal descriptions and the degree of subjective identification, using a scale ranging from −5 to 5.

### Experimental Procedure

The 50 participants were randomly divided into two groups. The experiment had a crossover design. On the first day of the experiment, the first group (25 people) viewed the fragrant Primula in the laboratory and experienced the olfactory stimulation, while the second group (25 people) viewed the odorless Primula in the laboratory, and thus did not receive olfactory stimulation. On the next day, the two groups were switched such that they repeated the experiment with the alternate condition. To avoid changes in measurement caused by the physiological clock, the activity took place from 8:00–11:30 am on each day.

Before the beginning of the experiment, the participants were informed regarding the content of the experiment and told that they could withdraw from the experiment at any time. All participants signed informed consent forms. First, the participants were invited into the preparation room and asked to sit down for 5 min. They then completed measurements of blood pressure, pulse rate, and completed a mood state scale. Subsequently, each participant entered the laboratory, put on EEG equipment, and closed their eyes to rest for 3 min to reach a stable state. Next, raw EEG data were acquired at a 1-min interval till the 10-min Primula exposure experience was completed. According to previous studies, 10 min experience was enough for the participants to have a significant recovery through exposure to the natural settings (Ulrich et al., 2002; Jo et al., 2013; Hassan et al., 2018a; Deng et al., 2020). After the experience, the participants rested for 5 min in a sitting position. They then underwent blood pressure and pulse rate measurements, and completed the mood state and semantic difference scales. When all measurements were completed, the participants were thanked for their involvement and excused from the laboratory. After the entire experiment was over, each participant would receive a small gift and a reward of $5.

### Data Analysis

Excel and SPSS 19 were used to analyze the statistical data. A paired *t*-test was used to analyze the pulse rate and blood pressure data. To analyze the EEG data, this study conducted a repeated measures ANOVA and performed the multivariate variance statistical test. The attention and relaxation scores were collected from the e-Sense™ software and analyzed using a one-way ANOVA. The SD data were also analyzed using a one-way ANOVA. The Wilcoxon signed rank test was used to analyze the POMS data. The threshold for statistical significance was set at *p* < 0.05.

## Results

### Blood Pressure and Pulse Rate

**Figure 2:**
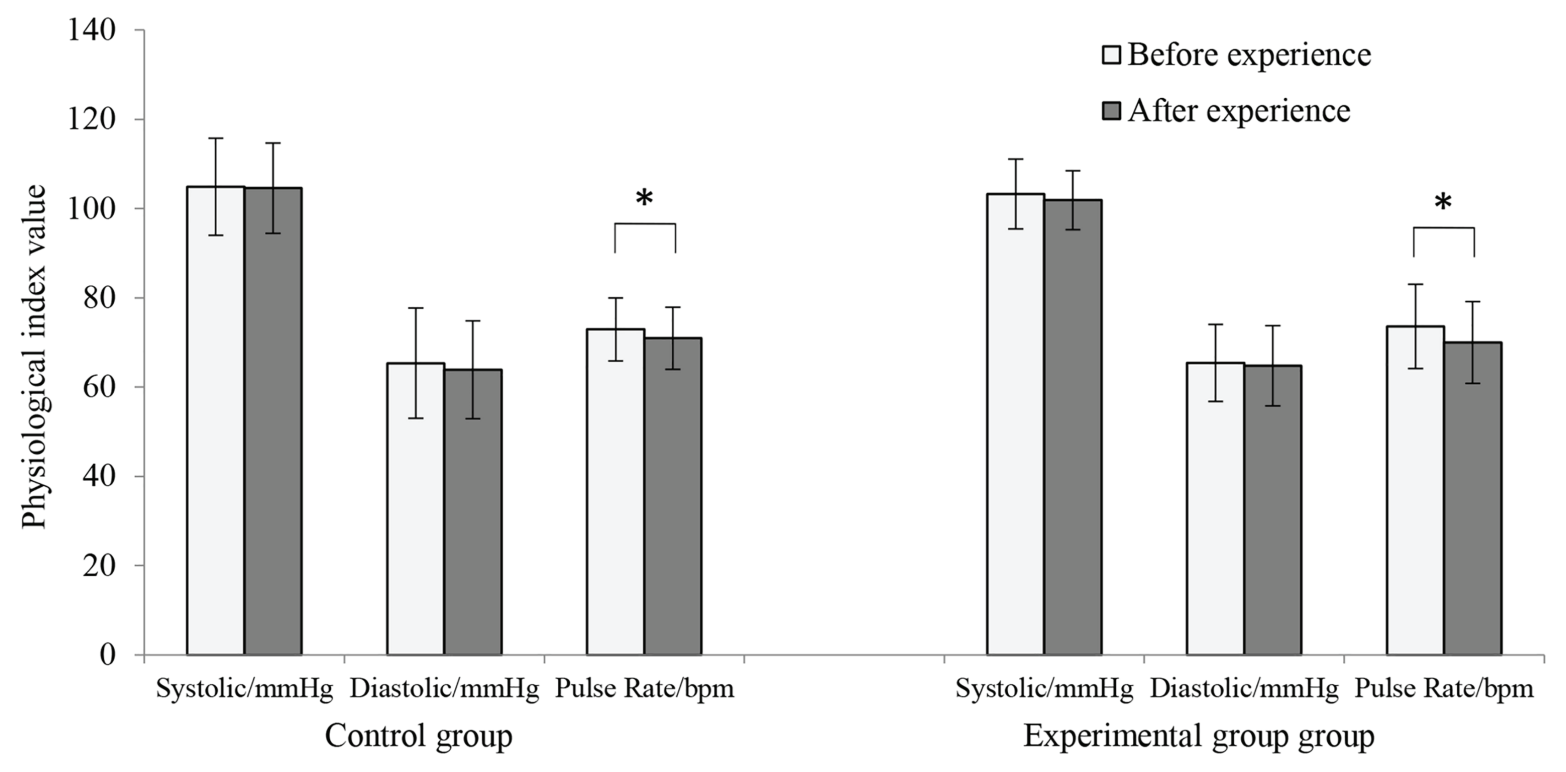
Systolic blood pressure, diastolic blood pressure, and pulse rate measurements before and after the experiment in the control condition and experimental condition (*n* = 50; mean ± SD; ^*^p < 0.05; verified by a paired *t*-test).

The systolic blood pressure, diastolic blood pressure, and pulse rate data are shown in Figure 2. Before the experiment, we found no significant differences between the control condition and experimental condition in terms of the three physiological indexes. After the experiment, the mean systolic blood pressure in the control condition exhibited a decreasing trend, although this difference was not significant (104.88 ± 10.90 and 104.56 ± 10.12, *p* > 0.05); the mean diastolic blood pressure exhibited a non-significant decreasing trend (65.34 ± 12.35 and 63.86 ± 10.97, *p* > 0.05); and the mean pulse rate was significantly decreased (72.92 ± 7.06 and 70.94 ± 6.99, *p* < 0.05).

After the participants completed the experimental condition, the mean systolic blood pressure exhibited a decreasing trend (103.24 ± 7.80 and 101.86 ± 6.57, *p* > 0.05), the mean diastolic blood pressure exhibited a decreasing trend (65.42 ± 8.65 and 64.76 ± 9.00, *p* > 0.05), and the mean heart rate was significantly decreased (73.56 + 9.44 and 70.02 + 9.17, *p* < 0.01).

Any differences between the experimental condition and control condition in terms of systolic blood pressure, diastolic blood pressure, and heart rate after the experiment were not significant.

### Brain Waves

**Figure 3:**
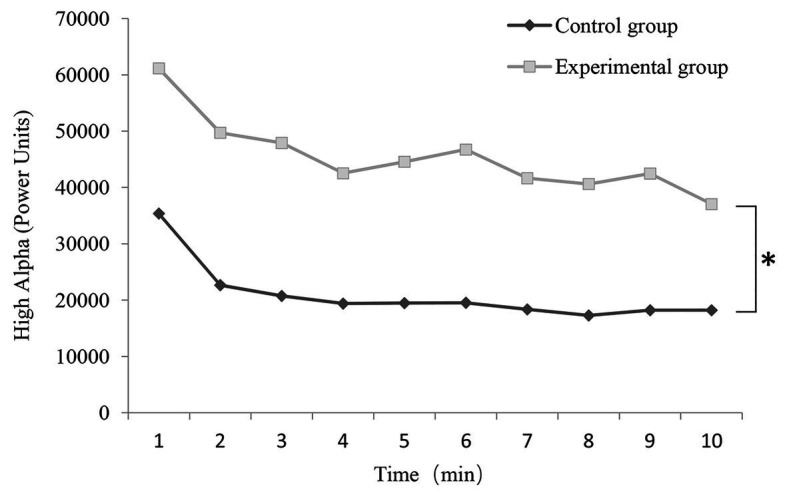
Change in high alpha wave (power units) value in each 1-min epoch in the control and experimental conditions (*n* = 50; mean ± SD; ^*^p < 0.05; verified by repeated ANOVA and multivariate variance statistical test).

**Figure 4:**
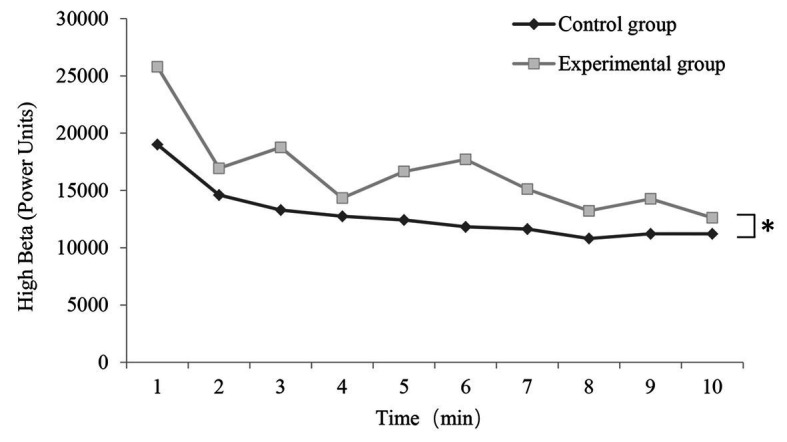
Change in high beta wave (power units) value in each 1-min epoch in the control and experimental conditions (*n* = 50; mean ± SD; ^*^p < 0.05; verified by repeated ANOVA and multivariate variance statistical test).

Figures 3, 4 show the EEG data. When we compared the high alpha and high beta EEG data before vs. after the experiment, we observed significant differences in both conditions. Analysis of the 1-min EEG epochs indicated that the mean alpha wave values and mean beta wave values were both higher when the participants completed the experimental vs. control condition. In each condition, the mean values of both the alpha and beta waves reached the highest point in the first minute of the experiment.

The alpha wave values in the control condition and experimental condition were analyzed by a repeated ANOVA, and the results are shown in Figure 3. The multivariate variance statistical test was performed based on Pillai’s Trace. As indicated from the result, mean alpha wave values between the conditions regarding changes over time showed a significant difference [*F*(9, 90) = 4.845, *p* = 0.000 < 0.05, *η*^2^ = 0.326]. No interaction was identified between time and condition [*F*(9, 90) = 0.912, *p* = 0.519 > 0.05, *η*^2^ = 0.084]. In addition, as suggested from Mauchly’s test of sphericity, *p* = 0.000 < 0.05, and the epsilon value was 0.490 < 0.750, so Greenhouse-Geisser was employed for correction (Maxwell and Delaney, 2004). The significant time effect of alpha wave was identified [*F*(4.410, 432.147) = 9.742, *p* = 0.000 < 0.05, *η*^2^ = 0.090], whereas a slight interaction was found between time and condition [*F*(4.410, 432.147) = 0.441, *p* = 0.797 > 0.05, *η*^2^ = 0.004]. The above results complied with those of the multivariate variance statistical test in the significance of time effect and interaction. The main effect of alpha wave values between the control condition and the experimental condition was significant [*F*(1, 98) = 32.171, *p* = 0.000 < 0.05, *η*^2^ = 0.247].

The beta wave values in the control condition and experimental condition were analyzed by a repeated ANOVA, and the results are shown in Figure 4. Pillai’s Trace was used in the multivariate variance statistical test, indicating that the beta wave varied significantly over time under both conditions [*F*(9, 90) = 6.981, *p* = 0.000 < 0.05, *η*^2^ = 0.410], and a slight interaction between time and condition was suggested [*F*(9, 90) = 1.252, *p* = 0.258 > 0.05, *η*^2^ = 0.117]. Moreover, as revealed in Mauchly’s test of sphericity, *p* = 0.000 < 0.05, and the value of epsilon reached 0.376 < 0.750, so Greenhouse-Geisser was employed for correction. A significant time effect of beta wave was identified [*F*(3.388, 332.035) = 13.370, *p* = 0.000 < 0.05, *η*^2^ = 0.120], whereas no interaction was reported between time and condition [F(3.388, 332.035) = 0.638, *p* = 0.610 > 0.05, *η*^2^ = 0.111]. The mentioned results complied with those of the multivariate variance statistical test in the significance of time effect and interaction. The main effect of beta wave values between the control condition and experimental condition was significant [*F*(1, 98) = 6.523, *p* = 0.012 < 0.05, *η*^2^ = 0.062].

**Figure 5:**
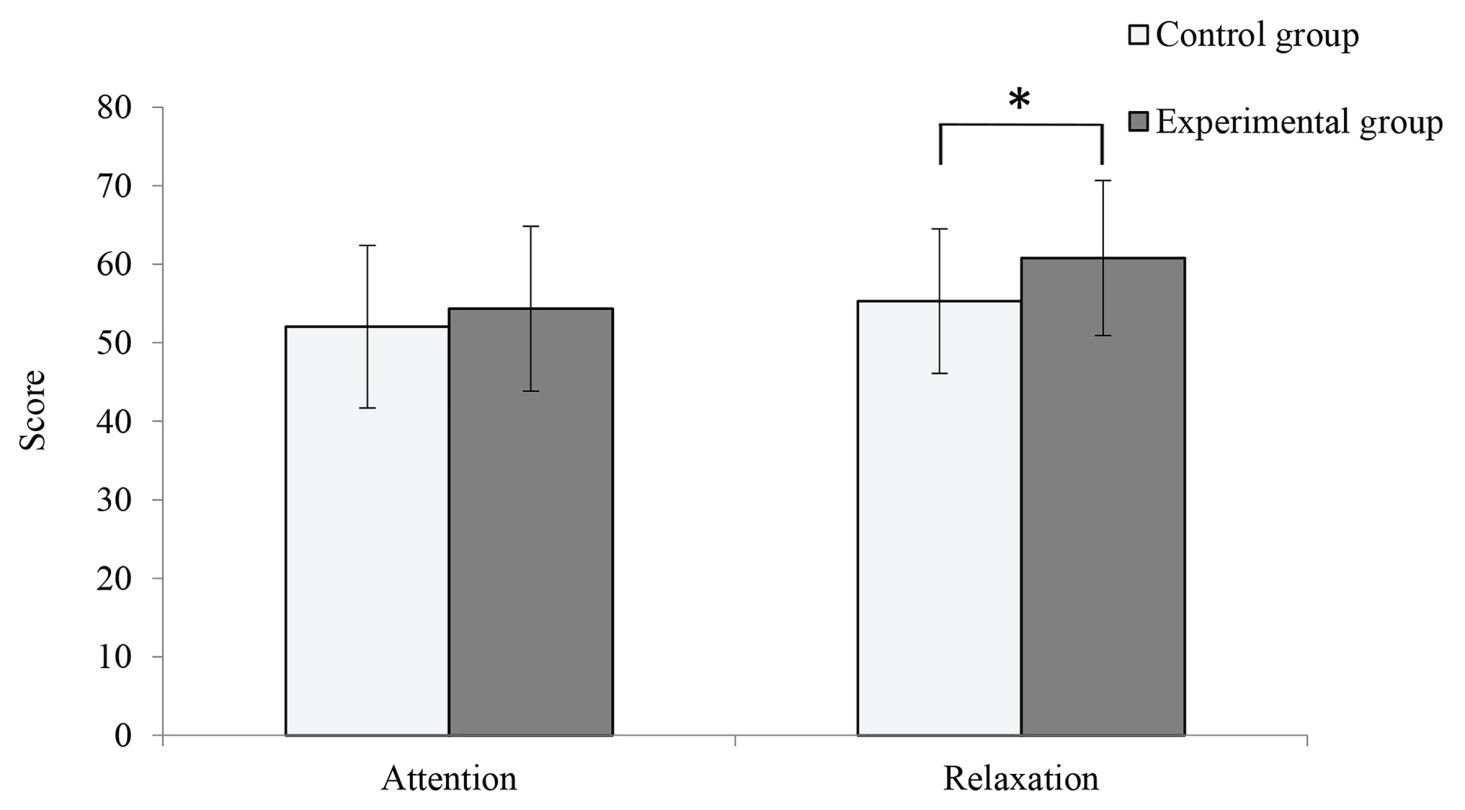
Comparison of mean attention and relaxation scores between the control and experimental conditions (*n* = 50; mean ± SD; ^*^p < 0.05; verified by one way ANOVA).

The results of the EEG analysis conducted using the e-Sense software are shown in Figure 5. The average attention score in the experimental condition was higher than that in the control condition (55.30 ± 9.18 and 52.04 ± 10.34, *p* > 0.05), and the average relaxation score in the experimental condition was significantly higher than that in the control condition (60.78 ± 9.87 and 54.34 ± 10.47, *p* < 0.05).

### Profile of Mood States

**Figure 6:**
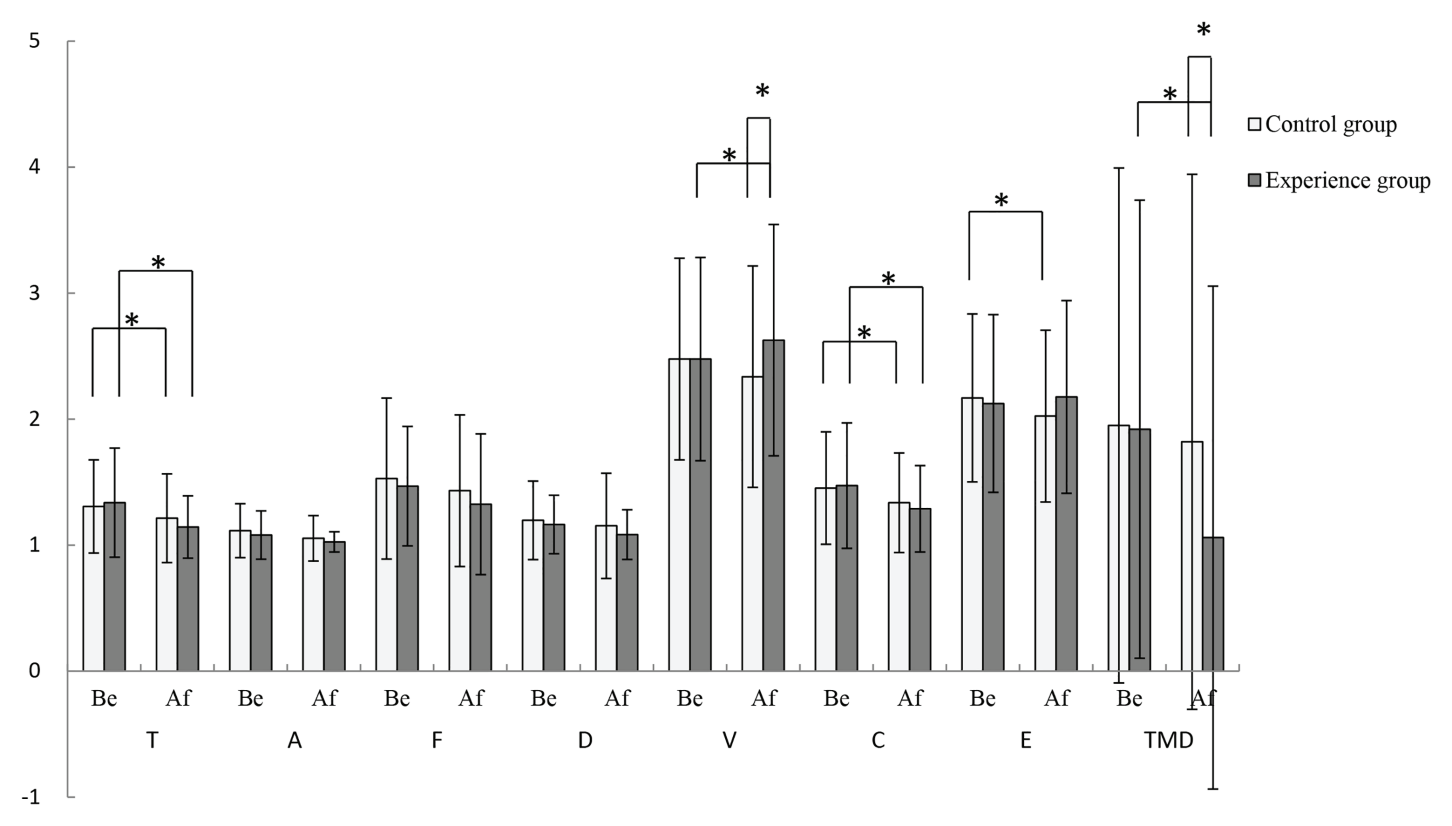
Profile of mood states (POMS) scores before and after the control condition and the experimental condition (*n* = 50; mean ± SD; ^*^p < 0.05; verified by a paired *t*-test).

The POMS results are shown in Figure 6. Before the experience, there were no significant differences between the control condition and the experimental condition. In the control condition, after viewing the non-fragrant Primula, the positive scale score increased, while the negative scale score decreased. The tension and confusion subscale scores decreased significantly, while self-esteem increased significantly. After the participants under the experimental condition observed and smelled the Primula, the scores for the respective scale were upregulated. To be specific, the average Tension, Confusion, TMD scores were downregulated significantly, and vitality score was elevated significantly. The subscale scores after experiencing the fragrant Primula were compared between the two conditions. The results showed that the average positive scale score was higher after the experimental condition vs. the control condition, and the average negative scale score was lower after the experimental condition vs. the control condition. The vitality subscale scores and the TMD after the experimental condition were significantly higher than those after the control condition, suggesting that the experimental condition induced greater vitality and was perceived as a better overall emotional experience compared with the control condition.

### SD Method

**Figure 7:**
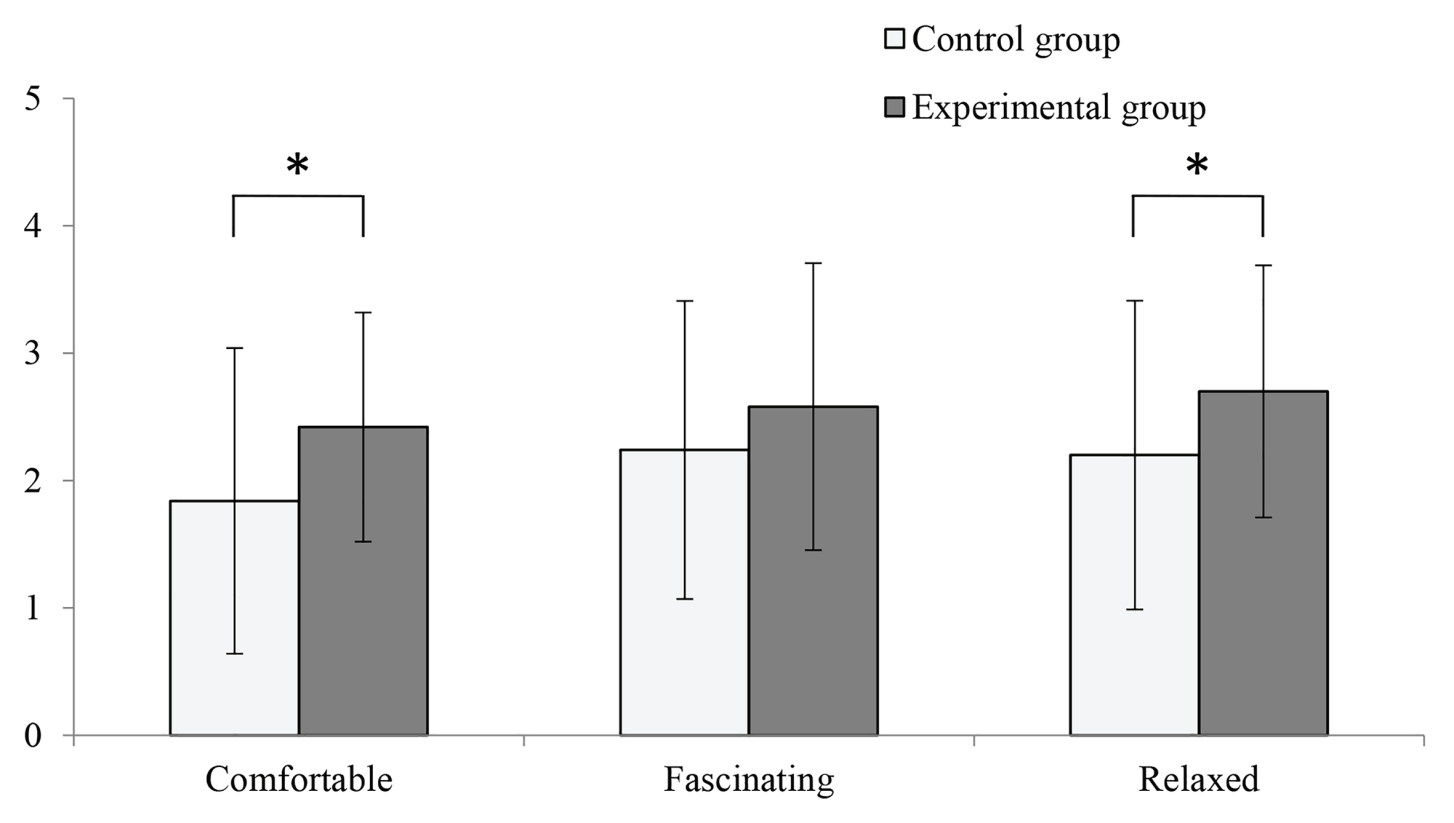
Comparisons between the control and experimental conditions, conducted using the semantic differential (SD) method (*n* = 50; mean ± SD; ^*^p < 0.05; verified by one-way ANOVA).

Figure 7 shows the differences in the participants’ subjective feelings, as measured using the SD method, according to exposure to the fragrant and non-fragrant Primula before and after the experiment. The mean values of “comfortable” and “relaxed” in the experimental condition were significantly higher than those in the control condition (comfortable: 1.84 ± 1.2 and 2.42 ± 0.94, *p* < 0.05; relaxed: 2.20 ± 1.21 and 2.70 ± 0.99, *p* < 0.05). The mean value of “fascinated” in the experimental condition was higher than that in the control condition, but this difference was not significant (2.24 ± 1.17 and 2.58 ± 1.12, *p* > 0.05).

## Discussion

### Effects on Blood Pressure and Pulse Rate

We observed a downward trend in the systolic blood pressure and diastolic blood pressure, and a significant decrease in the pulse rate in both the control and experimental conditions. Previous studies had shown that the sympathetic and parasympathetic nervous systems in the human body produce changes in blood pressure and heart rate when emotion is influenced by stress or relaxation (Schwartz et al., 1981; Deng and Deng, 2018). As mentioned in the literatures, the stress emotion induces the rise of blood pressure and pulse rate, while the relaxed emotion produces the decrease (Zanstra and Johnston, 2011; Hassan et al., 2018b). Therefore, it was presumably suggested that the Primula plants with or without fragrance could be able to regulate the sympathetic and parasympathetic nervous system and improve participants’ emotion. Our results supported previous studies on the emotional improvement caused by plants and plant fragrance (Lee et al., 2015; Zhao et al., 2019). Surprisingly, no significant differences were found in blood pressure and pulse rate between non-fragrant and fragrant Primula plants. This finding was also reported in the *Chrysanthemum indicum* (Kim et al., 2018). Whether Primula fragrance has additional effects on sympathetic nervous system and parasympathetic nervous system needs further study combined with more physiological indicators, such as high-frequency heart rate variability and galvanic skin response.

### Effects on EEG

Our results showed that the mean alpha wave value was significantly higher in the experimental condition compared with the control condition. In addition, the mean relaxation score in the experimental condition was significantly higher than that in the control condition. Alpha waves are known to be correlated with decreased mental stress, increased relaxation, and enhanced memory ability (Alarcao and Fonseca, 2017; Lina and Karwowski, 2020). Further, several reports have shown that exposure to the natural environment induces increased alpha wave strength, which is related to physiological relaxation and recovery effects (Kim et al., 2013, p. 38; Alarcao and Fonseca, 2017; Ursuţiu et al., 2018). This finding is consistent with that of Kim et al. (2016), who reported dramatically increased alpha waves after participants smelled an aromatic extract of orchid petals. Given the apparent connections between alpha wave power and relaxation (Hassan et al., 2018a; Tao et al., 2020), our findings, while preliminary, suggest that smelling fragrant Primula plants may have had a stronger restorative effect on participants than smelling non-fragrant Primula plants. Therefore, one of the suggestions of this result is to use fragrant flowers in places that need to create a relaxing atmosphere, such as studios, living rooms, hospitals, and so on.

Our results showed that both beta wave value and the attention score were significantly higher in the experimental condition compared with the control condition. Previous studies had shown that the emergence and modulation of beta waves represent attention and concentration (Coben et al., 2010). For instance, participants’ beta waves became stronger after viewing pictures of natural landscapes, and energy levels and attention increased (Jiang et al., 2019). This result is consistent with that of Hassan et al. (2018a), who reported significantly enhanced beta waves after viewing ornamental plants, while the participants became more attentive. These related studies showed that higher beta waves were associated with improved attention. In this study, the experimental condition produced higher beta value and attention score, suggesting that fragrant Primula may improve attention to a greater extent than non-fragrant Primula. Therefore, it may be suggested that an effective use of fragrant Primula is to be placed in the workplace, increase the concentration level of stuffs, and improve their working efficiency.

The mean alpha and beta values observed in the experimental condition were both significantly higher than those in the control condition, as were the attention and relaxation scores. This tends to infer that states of relaxation and concentration can occur simultaneously to some extent. These findings are consistent with related research on emotional changes caused by environmental contact, including virtual visual stimulation experiments (Guo et al., 2020), walking in a bamboo forest and a city environment (Hassan et al., 2018b), and horticultural activity (Hassan et al., 2018a), etc. These studies consistently reported higher mean alpha and beta values, reflecting relatively higher levels of relaxation and attention.

In terms of changes in EEG over time, the mean alpha and beta values in the two conditions reached their highest points during the first minute of the trial. Afterward, the mean alpha and beta values decreased and gradually reached a stable level. This may be due to changes in the experimental environment. Overall, the average alpha and beta values in the experimental condition were significantly higher than those in the control condition. Our results suggest that the fragrant Primula induced a stronger physiological and psychological effect, which reflects emotional relaxation and improved attention (Islarn and Ahmad, 2015). It suggested that olfaction benefited neural activity in a unique way, which could not be achieved by visual stimuli alone.

### Effects on Psychology

The results of the POMS questionnaire in the present study indicated trends toward improvement in most of the average scores on each scale in both the control and experimental conditions, with some significant changes. After the experiment, experimental condition got the higher positive scale score and the lower negative scale score vs. the control condition. Further, the mean TMD was significantly higher in the experimental vs. control condition, suggesting that the fragrant Primula induced a better emotional experience. The mean vitality scale score was significantly higher under the experimental condition than the control, demonstrating that aromatic stimulation of Primula can enable the participants to achieve more clear-headed and positive psychological state (Takayama et al., 2014). On a psychological level, fragrance is known to affect emotional state. Olfactory environments have been found to have an important impact on the spatial experience of residents and tourists, as well as playing a role in regulating psychology, relieving stress, and improving happiness and self-esteem (Glass et al., 2014). Therefore, the results of this study revealed the potential value of fragrant Primula in daily life space or public space, so as to promote people’s positive emotions and reduce negative emotions.

The SD directly reflects the subjective feelings of respondents. In this study, the SD data likely indicated that the fragrant Primula made the participants feel more relaxed and comfortable. In related studies, fresh roses were used as an olfactory stimulant, and the results of the SD also showed that participants exposed to the roses experienced a heightened sense of comfort compared with a control condition (Igarashi et al., 2014). Therefore, the results of this study were consistent with previous studies.

### Relationship Between Physiological and Psychological Findings

After examining the collected physiological and psychological data, we were able to infer that the changes in these two data types were related. After contact with two kinds of Primula, the participant blood pressure and pulse had decreased by varying degrees. Further, scores on the positive subscales in the POMS had increased, and scores on the negative subscales had decreased. These data inferred that both kinds of Primula elicited some degree of improvement in physiological and psychological state (Park et al., 2017).

Our finding of significantly higher alpha values and relaxation scores in the experimental condition compared with the control condition confirmed that the fragrant Primula induced a more powerful relaxation effect. The results of POMS and SD showed that participants in the experimental condition had lower TMD scores and higher relaxation scores, which is consistent with the EEG data. The fragrant Primula induced higher beta values, which corresponded to the significant increase in the average vigor scale score in the POMS. Taken together, these physiological and psychological results support each other, which may infer that the fragrant Primula made the participants more relaxed and more attentive, which likely had a positive effect on emotions.

In general, both kinds of Primula improved the physiological and psychological state of participants, although the fragrant Primula had a stronger effect.

### Research Limitations

This study had the following limitations, which should be taken into account in future research. First, as impacted by the limitations of the experiment scale, only female college students were included here as participants. Future studies should examine people with different demographic characteristics and cross-cultural backgrounds to explore the universality of the experimental results. Second, we only studied the effects of aromatic plant stimulation from several physiological and psychological aspects, while measurements in additional fields, such as pharmacology, biomedicine, and cognitive psychology, may be informative. Therefore, future studies should involve a more diverse participant group, use the evaluation of more physiological indexes, and complete a more in-depth exploration of the specific mechanisms of olfactory effects.

## Conclusion

In this study, we used blood pressure, pulse rate, EEG, POMS, and SD data to examine the effects of the fragrant *Primula forbesii Franch* and the non-fragrant *Primula malacoides Franch* on the physiological and psychological state of female college students. Compared with the non-fragrant Primula, the fragrant Primula modulated EEG and many psychological indexes in a way that reflected greater relaxation and attention. This infers that, in addition to the positive impact of visual stimulation, aromatic stimulation *via* the Primula plant can have important beneficial effects.

Our results suggest that indoor plants, which are a simple and economic way to improve the quality of indoor environments, have beneficial health effects and are thus worth promoting. Further, our data demonstrate the important role of aromatic stimulation in the effects of indoor plants on human health. Therefore, we suggest that aromatic plants be selected during indoor plant landscaping to better promote the physical and psychological health of visitors, and improve quality of life. This notion is especially worth promoting in areas where public green areas are scarce.

## Data Availability Statement

The raw data supporting the conclusions of this article will be made available by the authors, without undue reservation.

## Ethics Statement

The studies involving human participants were reviewed and approved by local Ethics Committee of the College of Landscape Architecture, Sichuan Agricultural University, China. The patients/participants provided their written informed consent to participate in this study.

## Author Contributions

XL, YJ, and LD contributed to conception of the study. LD and MJ contributed to design of the study. SJ and LD contributed to statistical analysis. LD, HL, BG, and YJ contributed to experimental organization. SJ wrote the first draft of the manuscript. SJ, XL, HL, BG, MJ, YJ, JM, LS, and ZH reviewed and edited the draft. SJ, XL, and MJ contributed to project administration. All authors contributed to the article and approved the submitted version.

### Conflict of Interest

The authors declare that the research was conducted in the absence of any commercial or financial relationships that could be construed as a potential conflict of interest.
